# Supplementary material for: Transfer of disulfide bond formation modules via yeast artificial chromosomes promotes the expression of heterologous proteins in Kluyveromyces marxianus
Source: mLife. 2024 Mar 22;3(1):129–42. doi: 10.1002/mlf2.12115 (PMC11139206; doi:10.1002/mlf2.12115)
Supplement: Supplementary file 4 — Supporting information. [file MLF2-3-129-s005.docx]

LOCUS LHZ1015 10276 bp DNA circular 23-FEB-2022

SOURCE

ORGANISM

COMMENT This file is created by Vector NTI

http://www.invitrogen.com/

COMMENT VNTDATE|-14275849|

COMMENT VNTDBDATE|-14277271|

COMMENT LSOWNER|

COMMENT VNTNAME|LHZ1015|

COMMENT VNTAUTHORNAME|Demo User|

COMMENT VNTOAUTHORNAME|Yao Yu|

FEATURES Location/Qualifiers

CDS complement(9216..10076)

/vntifkey="4"

/label=AmpR

rep_origin 8457..9045

/vntifkey="33"

/label=Rep(pMB1)

CDS 1893..3138

/vntifkey="4"

/label=ARS1

promoter 3139..3517

/vntifkey="29"

/label=TEF\Promoter

CDS 3518..4552

/vntifkey="4"

/label=HphMX4

terminator 4553..4787

/vntifkey="43"

/label=TEF\terminator

CDS 5348..6013

/vntifkey="4"

/label=TRP1\CDS

promoter 4788..5347

/vntifkey="29"

/label=TRP1\promoter

terminator 6014..6207

/vntifkey="43"

/label=TRP1\Terminator

CDS 1021..1686

/vntifkey="4"

/label=HIS3\CDS

promoter 419..1020

/vntifkey="29"

/label=HIS3\Promoter

terminator 1687..1886

/vntifkey="43"

/label=HIS3\Terminator

misc_feature 6208..6841

/vntifkey="21"

/label=KM\Telomere

CDS 6858..7357

/vntifkey="4"

/label=5'Abe-GLA

misc_feature complement(7366..7999)

/vntifkey="21"

/label=KM\Telomere

BASE COUNT 2881 a 2294 c 2243 g 2858 t

ORIGIN

1 tcgcgcgttt cggtgatgac ggtgaaaacc tctgacacat gcagctcccg gagacggtca

61 cagcttgtct gtaagcggat gccgggagca gacaagcccg tcagggcgcg tcagcgggtg

121 ttggcgggtg tcggggctgg cttaactatg cggcatcaga gcagattgta ctgagagtgc

181 accatatgcg gtgtgaaata ccgcacagat gcgtaaggag aaaataccgc atcaggcgcc

241 attcgccatt caggctgcgc aactgttggg aagggcgatc ggtgcgggcc tcttcgctat

301 tacgccagct ggcgaaaggg ggatgtgctg caaggcgatt aagttgggta acgccagggt

361 tttcccagtc acgacgttgt aaaacgacgg ccagtgaatt gacgcgtatt gggatatcag

421 aatttttgtc tcttaccaaa aggatctacg tcaccattta aagccctttc agtcttctgc

481 ttcgcgtgca catgagaata tttcttctta aaccactcgt ccttatccat gccatctttt

541 tcccatgctt tcgttctctt ttgtatcggg aagttccttc caaatgttac tttcttgtct

601 ttgatgaagg attcatttgg gattttcacc gctggacgaa attctggtgg caaagcctct

661 ataaacagct ttcatgatat ttattgttga caacttaata ttactgttaa gaaaaggcgg

721 atgagaatac ctagctctct agtatctttc taatagatat accaagctca cattatctca

781 actcatctca tctcatctct atactgcaac tttttcttta gaaatttttc aagaagtgat

841 attttcttag acattttttt ttttttttaa aaggctccaa gaatgattca taatatgaaa

901 tcaagtataa atgcagctgt acaatggttt aacttagttt aagatccgtt cggaagtact

961 attattgtaa cattttacac aataagaagg tcttcaaata aggactttga taccgagaca

1021 atgacatacc cagaaaggaa ggcttttgtg tctagaataa caaatgagac aaaaattcag

1081 atagccatat ccttacatgg ggggcatatc tcaatcccaa attctatact ggatagaccg

1141 gagtcggacg ttgcaaaaca agctactggt tcacagatta ttgacattca aactggtatt

1201 ggatttctag atcatatgat tcatgcccta gcgaagcact ctggttggtc cttaattgtc

1261 gaatgtatcg gagatttgca tatcgatgac caccatacta cggaggattg tggtattgct

1321 ctaggacagg cttttaaaga agcattaggt catgtccgtg gtgtgagaag atttggtact

1381 ggatttgcac cattggacga agcattatca agggccgtcg ttgatctatc caacagacca

1441 ttcgccgtaa tagatttggg tttaaaaaga gaaaaaatcg gtgatctttc atgtgaaatg

1501 ataccacatt tcttggagtc atttgcggaa gctgcaagag taactttaca tgttgactgt

1561 ttaagaggct ttaatgatca tcacagaagt gagtcagcct ttaaggctct tgctgtggct

1621 atcagagagg ctatttccag taacggtaca aatgacgttc catctaccaa aggagttttg

1681 atgtaactgg ctactccata caaggcggta taaataaaat ataataacat gtttgtacaa

1741 taatgttttc ctatttatta cttcatatat tatatatgtt gcacctaaaa taaggaagtt

1801 attctcaaag ttattgtgta cttttatatt atttacatgg gaatttatat atatatatat

1861 gtgcgtgtgg tgacagcata acgcccctcg agatcgattg aagttttgtc caactatcca

1921 ctatggatat gcgttttgtt gattaacctt aaataacacg tatttcgcat tttccaaaag

1981 ccttttttca taactacaaa ctaactattt ttgtttattt tacatcagta aattatgcgc

2041 agaaaatatg taaggctata tactcaatat agtggaagag cctctggcta cttaatctgg

2101 gttcatatat tctgtcagtg gtatagtaaa tttaaatagt gaatttgggc gcatgtagag

2161 agatcttttg attaataacc cagtatttga ttcttgaatg ttatttgtct cttattaagt

2221 atttcagttt gatttatata ttttgaaagt aattgttgct ttaccttcca aacaataaaa

2281 aaatataaaa aaatgtaaaa aatataataa atattaaata aaatactact tgtttgaaaa

2341 tcagagaaaa tccaccaaaa tatcaatcat ttcaaggatt tccgaaccaa gttcgagata

2401 gtcctttaag gtcagtaaaa ttcagttgca cgtataacag atatttttca ttttgttcca

2461 atttaaaagt cccccatttt taaaattatt gaaaataaaa attaaaaaat taaaaggaat

2521 ctctctatgt cactttaaaa taaataaatt gaaaatgata tatcgttaaa agtcaaccgc

2581 aacaccacct atttctaaga ggagagttct aaaaaaatca tagtaccaca caggtaaact

2641 aaaacagcta aattcaacat aatacctgtg gatataatta catacaaaaa tataattaaa

2701 aaaatacatt aaaaataatt tatttttgta aaacccataa aatatatttt actttcggaa

2761 caacttttta acttataatt ttgttttaaa taaaaacgtt gtatttaaaa ataataaaat

2821 attaagtaaa aatttaaagc atatttattt aaaatataaa atactactaa aattactcta

2881 aacttcaaaa taaaaaaata ataaattata tagttttaaa attaataatt tgtatacacg

2941 tgaccagacc ataatagttt tcttttcttg aaactgccat gaatttaata gatttttttt

3001 acacatattt acagtaagtt ttgtttttac gttgaattaa tattgtatac gtacttagaa

3061 ggataacttc caactatgaa tatgtaggtt aaaaggtaaa tagagaagcc ttacttttat

3121 cagaaatgaa aggagctcag cttgccttgt ccccgccggg tcacccggcc agcgacatgg

3181 aggcccagaa taccctcctt gacagtcttg acgtgcgcag ctcaggggca tgatgtgact

3241 gtcgcccgta catttagccc atacatcccc atgtataatc atttgcatcc atacattttg

3301 atggccgcac ggcgcgaagc aaaaattacg gctcctcgct gcagacctgc gagcagggaa

3361 acgctcccct cacagacgcg ttgaattgtc cccacgccgc gcccctgtag agaaatataa

3421 aaggttagga tttgccactg aggttcttct ttcatatact tccttttaaa atcttgctag

3481 gatacagttc tcacatcaca tccgaacata aacaaccatg ggtaaaaagc ctgaactcac

3541 cgcgacgtct gtcgagaagt ttctgatcga aaagttcgac agcgtctccg acctgatgca

3601 gctctcggag ggcgaagaat ctcgtgcttt cagcttcgat gtaggagggc gtggatatgt

3661 cctgcgggta aatagctgcg ccgatggttt ctacaaagat cgttatgttt atcggcactt

3721 tgcatcggcc gcgctcccga ttccggaagt gcttgacatt ggggaattca gcggccgcga

3781 gagcctgacc tattgcatct cccgccgtgc acagggtgtc acgttgcaag acctgcctga

3841 aaccgaactg cccgctgttc tgcagccggt cgcggaggcc atggatgcga tcgctgcggc

3901 cgatcttagc cagacgagcg ggttcggccc attcggaccg caaggaatcg gtcaatacac

3961 tacatggcgt gatttcatat gcgcgattgc tgatccccat gtgtatcact ggcaaactgt

4021 gatggacgac accgtcagtg cgtccgtcgc gcaggctctc gatgagctga tgctttgggc

4081 cgaggactgc cccgaagtcc ggcacctcgt gcacgcggat ttcggctcca acaatgtcct

4141 gacggacaat ggccgcataa cagcggtcat tgactggagc gaggcgatgt tcggggattc

4201 ccaatacgag gtcgccaaca tcttcttctg gaggccgtgg ttggcttgta tggagcagca

4261 gacgcgctac ttcgagcgga ggcatccgga gcttgcagga tcgccgcggc tccgggcgta

4321 tatgctccgc attggtcttg accaactcta tcagagcttg gttgacggca atttcgatga

4381 tgcagcttgg gcgcagggtc gatgcgacgc aatcgtccga tccggagccg ggactgtcgg

4441 gcgtacacaa atcgcccgca gaagcgcggc cgtctggacc gatggctgtg tagaagtact

4501 cgccgatagt ggaaaccgac gccccagcac tcgtccgagg gcaaaggaat aatcagtact

4561 gacaataaaa agattcttgt tttcaagaac ttgtcatttg tatagttttt ttatattgta

4621 gttgttctat tttaatcaaa tgttagcgtg atttatattt tttttcgcct cgacatcatc

4681 tgcccagatg cgaagttaag tgcgcagaaa gtaatatcat gcgtcaatcg tatgtgaatg

4741 ctggtcgcta tactgctgtc gattcgatac taacgccgcc atccagtggt accgtcgcct

4801 tgagagaaat tagaagattc caaaaatcca ccgaactatt gatcagaaag ttgcctttcc

4861 aaagattggt tagagaaatc gcccaagact tcaagaccga tttgagattc caatcttctg

4921 ctatcggtgc cttgcaagaa tccgtcgaag cctacttggt ctccttgttc gaagacacca

4981 acttggctgc catccacgcc aagagagtca ccatccaaaa gaaggacatc aagttcgaag

5041 acaccaactt ggctgccatc cacgccaaga gagtcaccat ccaaaagaag gacatcaagt

5101 tggccagaag attgagaggt gagagatcgt gaatgttctt ttccccttcc tttccccttc

5161 ccttctaatt ttatctttat tatctactta ttttactggc tagtccagtt tttttcaacg

5221 cttcttttcc cctaggaaaa aatagagaag cgcaataagt atatcccagg tgtataatag

5281 tttaatatca atcgagtaat atacggtttt ctcaggaata acaactgcct tagtctagtc

5341 cacagacatg ctcgtcaaga tctgcggctt gcagtctgtt gaagctgctc aaacagcgct

5401 ggatcgcggc gcagacctgc tgggagtcat atgtgtcccc aacaggaaac gcaccgtcac

5461 gccagcaaca gcaaaacaaa tctcacaact ggttcaccag ggcaatcatt cccaggggaa

5521 tcaccaggcc aggctggtcg gggtgttccg gaaccagcct ctagaagaag tgctcgccct

5581 gtaccacgaa tacaacctag acgttatcca gcttcacggc aacgaagatg tggtccaatg

5641 gagaaaatgg attcccaagg acatcacgtt gatcaaggcg ttccagttcc ctggcgactg

5701 cgacgtggtg ttatcgccag cggtcgccca gctccagctg gaaaacgtgc tggtgctgtt

5761 cgattcgggc gaaggtggca cgggccagca gctcgactgg aacggcatgg ccagctggtg

5821 tcagaaccaa ggtactacca cccgcttcat actcgcggga ggactcaccc cagataacgt

5881 gggccacgcc atcacaagcc tcgcgcccca tgccatcgga gttgacgtca gcggaggtgt

5941 cgagacaaac ggccagaagg acatggccaa gatcgccgcc ttcatatcac aggcgagggg

6001 tctatctatc taataagtaa gtagagcatt ggttaatgaa tacataggta ataattactg

6061 tatttcctct agctagtacc ccgcagctca agcagaaccg gagatgaaga accacttgtc

6121 aatggacttg tcgattggct cgtctggcaa gacgtgtgct ggtggaacag cagcagcagc

6181 ttcggcagag taagtggcag tgtcgacggt gtacggattt gattagttat gtggtgtacg

6241 gatttgatta gttatgtggt gtacggattt gattagttat gtggtgtacg gatttgatta

6301 gttatgtggt gtacggattt gattagttat gtggtgtacg gatttgatta gttatgtggt

6361 gtacggattt gattagttat gtggtgtacg gatttgatta gttatgtggt gtacggattt

6421 gattagttat gtggtgtacg gatttgatta gttatgtggt gtacggattt gattagttat

6481 gtggtgtacg gatttgatta gttatgtggt gtacggattt gattagttat gtggtgtacg

6541 gatttgatta gttatgtggt gtacggattt gattagttat gtggtgtacg gatttgatta

6601 gttatgtggt gtacggattt gattagttat gtggtgtacg gatttgatta gttatgtggt

6661 gtacggattt gattagttat gtggtgtacg gatttgatta gttatgtggt gtacggattt

6721 gattagttat gtggtgtacg gatttgatta gttatgtggt gtacggattt gattagttat

6781 gtggtgtacg gatttgatta gttatgtggt gtacggattt gattagttat gtggtgtacg

6841 ggcggatccg caagcttttc caagttagat tcaagccttc cgaggacacc gccttagaca

6901 ctgtcgatga cggcaccttg cagtccttgt tggacaacat cggcttgaac ggttctaatg

6961 cttgggacac cagaccgggt ttggttatcg cctccccttc caagaaggac cctaactact

7021 tcttcacttg gactcgtgac tccgccttgg tcttaaaatg tatcaccgac gctttcgctg

7081 ctggtaatac cgccttgcaa gaaaccatcc acgagtacat ctcttcccaa gctcgtatcc

7141 agttattgaa caccagatcc ggcggtttgt cctccggcgg tttaggcgag ccaaagtacc

7201 gtgttgacga gaccccttac aacgaagatt ggggtagacc acaagctgat ggtccagcct

7261 tgcgtgccac tgccttgatt gcttacgccc gttggttatt ggaaaatgac tactacgacg

7321 ttgccaagtc tatcgtttgg ccagttgtta agaacgagga tccgcccgta caccacataa

7381 ctaatcaaat ccgtacacca cataactaat caaatccgta caccacataa ctaatcaaat

7441 ccgtacacca cataactaat caaatccgta caccacataa ctaatcaaat ccgtacacca

7501 cataactaat caaatccgta caccacataa ctaatcaaat ccgtacacca cataactaat

7561 caaatccgta caccacataa ctaatcaaat ccgtacacca cataactaat caaatccgta

7621 caccacataa ctaatcaaat ccgtacacca cataactaat caaatccgta caccacataa

7681 ctaatcaaat ccgtacacca cataactaat caaatccgta caccacataa ctaatcaaat

7741 ccgtacacca cataactaat caaatccgta caccacataa ctaatcaaat ccgtacacca

7801 cataactaat caaatccgta caccacataa ctaatcaaat ccgtacacca cataactaat

7861 caaatccgta caccacataa ctaatcaaat ccgtacacca cataactaat caaatccgta

7921 caccacataa ctaatcaaat ccgtacacca cataactaat caaatccgta caccacataa

7981 ctaatcaaat ccgtacaccg tcgacctgca ggcatgcaag cttccaatgg cgcgccgagc

8041 ttggcgtaat catggtcata gctgtttcct gtgtgaaatt gttatccgct cacaattcca

8101 cacaacatac gagccggaag cataaagtgt aaagcctggg gtgcctaatg agtgagctaa

8161 ctcacattaa ttgcgttgcg ctcactgccc gctttccagt cgggaaacct gtcgtgccag

8221 ctgcattaat gaatcggcca acgcgcgggg agaggcggtt tgcgtattgg gcgctcttcc

8281 gcttcctcgc tcactgactc gctgcgctcg gtcgttcggc tgcggcgagc ggtatcagct

8341 cactcaaagg cggtaatacg gttatccaca gaatcagggg ataacgcagg aaagaacatg

8401 tgagcaaaag gccagcaaaa ggccaggaac cgtaaaaagg ccgcgttgct ggcgtttttc

8461 cataggctcc gcccccctga cgagcatcac aaaaatcgac gctcaagtca gaggtggcga

8521 aacccgacag gactataaag ataccaggcg tttccccctg gaagctccct cgtgcgctct

8581 cctgttccga ccctgccgct taccggatac ctgtccgcct ttctcccttc gggaagcgtg

8641 gcgctttctc atagctcacg ctgtaggtat ctcagttcgg tgtaggtcgt tcgctccaag

8701 ctgggctgtg tgcacgaacc ccccgttcag cccgaccgct gcgccttatc cggtaactat

8761 cgtcttgagt ccaacccggt aagacacgac ttatcgccac tggcagcagc cactggtaac

8821 aggattagca gagcgaggta tgtaggcggt gctacagagt tcttgaagtg gtggcctaac

8881 tacggctaca ctagaagaac agtatttggt atctgcgctc tgctgaagcc agttaccttc

8941 ggaaaaagag ttggtagctc ttgatccggc aaacaaacca ccgctggtag cggtggtttt

9001 tttgtttgca agcagcagat tacgcgcaga aaaaaaggat ctcaagaaga tcctttgatc

9061 ttttctacgg ggtctgacgc tcagtggaac gaaaactcac gttaagggat tttggtcatg

9121 agattatcaa aaaggatctt cacctagatc cttttaaatt aaaaatgaag ttttaaatca

9181 atctaaagta tatatgagta aacttggtct gacagttacc aatgcttaat cagtgaggca

9241 cctatctcag cgatctgtct atttcgttca tccatagttg cctgactccc cgtcgtgtag

9301 ataactacga tacgggaggg cttaccatct ggccccagtg ctgcaatgat accgcgagac

9361 ccacgctcac cggctccaga tttatcagca ataaaccagc cagccggaag ggccgagcgc

9421 agaagtggtc ctgcaacttt atccgcctcc atccagtcta ttaattgttg ccgggaagct

9481 agagtaagta gttcgccagt taatagtttg cgcaacgttg ttgccattgc tacaggcatc

9541 gtggtgtcac gctcgtcgtt tggtatggct tcattcagct ccggttccca acgatcaagg

9601 cgagttacat gatcccccat gttgtgcaaa aaagcggtta gctccttcgg tcctccgatc

9661 gttgtcagaa gtaagttggc cgcagtgtta tcactcatgg ttatggcagc actgcataat

9721 tctcttactg tcatgccatc cgtaagatgc ttttctgtga ctggtgagta ctcaaccaag

9781 tcattctgag aatagtgtat gcggcgaccg agttgctctt gcccggcgtc aatacgggat

9841 aataccgcgc cacatagcag aactttaaaa gtgctcatca ttggaaaacg ttcttcgggg

9901 cgaaaactct caaggatctt accgctgttg agatccagtt cgatgtaacc cactcgtgca

9961 cccaactgat cttcagcatc ttttactttc accagcgttt ctgggtgagc aaaaacagga

10021 aggcaaaatg ccgcaaaaaa gggaataagg gcgacacgga aatgttgaat actcatactc

10081 ttcctttttc aatattattg aagcatttat cagggttatt gtctcatgag cggatacata

10141 tttgaatgta tttagaaaaa taaacaaata ggggttccgc gcacatttcc ccgaaaagtg

10201 ccacctgacg tctaagaaac cattattatc atgacattaa cctataaaaa taggcgtatc

10261 acgaggccct ttcgtc

//
